# N, S and Transition-Metal Co-Doped Graphene Nanocomposites as High-Performance Catalyst for Glucose Oxidation in a Direct Glucose Alkaline Fuel Cell

**DOI:** 10.3390/nano11010202

**Published:** 2021-01-14

**Authors:** Yexin Dai, Jie Ding, Jingyu Li, Yang Li, Yanping Zong, Pingping Zhang, Zhiyun Wang, Xianhua Liu

**Affiliations:** 1 Tianjin Key Laboratory of Indoor Air Environmental Quality Control, School of Environmental Science and Engineering, Tianjin University, Tianjin 300354, China; daiyexinf@163.com (Y.D.); aichachajun@foxmail.com (J.D.); jingyuli@tju.edu.cn (J.L.); liyang234@tju.edu.cn (Y.L.); 2 Tianjin Marine Environmental Center Station, State Oceanic Administration, Tianjin 300450, China; zongyanping@bhfj.gov.cn; 3 College of Food Science and Engineering, Tianjin Agricultural University, Tianjin 300384, China; zpp613@163.com

**Keywords:** reduced graphene oxide, N, S, transitional metal, doping, direct glucose alkaline fuel cell

## Abstract

In this work, reduced graphene oxide (rGO) nanocomposites doped with nitrogen (N), sulfur (S) and transitional metal (Ni, Co, Fe) were synthesized by using a simple one-step in-situ hydrothermal approach. Electrochemical characterization showed that rGO-NS-Ni was the most prominent catalyst for glucose oxidation. The current density of the direct glucose alkaline fuel cell (DGAFC) with rGO-NS-Ni as the anode catalyst reached 148.0 mA/cm^2^, which was 40.82% higher than the blank group. The DGAFC exhibited a maximum power density of 48 W/m^2^, which was more than 2.08 folds than that of blank group. The catalyst was further characterized by SEM, XPS and Raman. It was speculated that the boosted performance was due to the synergistic effect of N, S-doped rGO and the metallic redox couples, (Ni^2+^/Ni^3+^, Co^2+^/Co^3+^ and Fe^2+^/Fe^3+^), which created more active sites and accelerated electron transfer. This research can provide insights for the development of environmental benign catalysts and promote the application of the DGAFCs.

## 1. Introduction

The energy crisis and environmental pollution have become two major issues worldwide in the 21st century [1,2,3]. The development of novel green and sustainable energy technologies to reduce the negative impact on the environment is of vital importance to human beings. Many countries are investing heavily in the development and utilization of clean energy to replace traditional fossil energy. Compared with wind energy and solar energy, the fuel cell has characteristics of simple conversion, portability and low volatility. It has been widely considered by the scientific community as an efficient alternative to fossil fuel. The fuel cell is a power generation device that directly converts the chemical energy of fuel into electrical energy through an electrochemical reaction without combustion. The traditional internal combustion engine has an efficiency of only about 35–40%, while the fuel cell was unlimited by the Carnot cycle. The theoretical efficiency can reach more than 80%. It was considered to be one of the most promising new chemical power sources.

Several types of substances were used as fuel for power generation, such as hydrogen [4], methanol [5], formic acid [6], glycerin [7] and glucose [8]. Glucose was the most abundant monosaccharide in nature and can be easily produced from biomass [9]. It has the characteristics of low cost, high production and being sustainable. The complete oxidation of a molecule of glucose to CO_2_ can release 24 electrons and generate 2.87 × 10^6^ J/mol of energy. The direct glucose alkaline fuel cell (DGAFC) has the advantages of simple structure, high stability and strong durability, which has gained wide attention all over the world. However, due to the low efficiency of glucose oxidation, the strategy of directly making full use of glucose in fuel cells was imperfect. Therefore, it was significant to develop an environmentally friendly, economical and excellent catalyst to improve the performance and stability of DGAFC. Noble metal catalysts (platinum, ruthenium, palladium) and precious metal alloys were considered to be the most effective electrocatalysts [10,11,12], but there were bottlenecks such as high cost and easy poisoning. A large number of studies have shown that transition metals (Ni Co Fe Cu) and their oxides (NiO, Co_3_O_4_ and CuO) exhibit good electrocatalytic activity and become a substitute for precious metals. Sarwar et al. [13] prepared a Co-Ni/graphene composite catalyst and tested it in a methanol fuel cell. It is found that increasing the concentration of nickel in the composite catalyst can significantly promote the oxidation reaction of methanol. Dong et al. [14] used FeCoO_4_ composite catalysts in direct glucose fuel cells and achieved a maximum power density of 35.91 W/m^2^. Zhao et al. [15] prepared high-performance graphene oxide and nickel oxide modified foam nickel electrodes, and achieved a maximum power density of 13.48 W/m^2^ in DGAFC, which was 39.3% higher than the blank group. In addition to the catalytic activity of the catalyst itself, the catalyst carrier determines the active surface area and electrode conductivity, which helps to disperse the catalyst particles and has a synergistic effect with the catalyst, which plays a very important role in improving the performance of the electrode. Carbon-based materials such as glassy carbon, high-order pyrolytic graphite, carbon nanotubes and graphene are commonly used as support materials for electrocatalysts.

Reduced graphene oxide (rGO) is a new type of monoatomic layer nanomaterial with good strength, flexibility and conductivity. Some reports showed that the electronic structure and chemical reaction properties of rGO-based materials can be improved by the introduction of vacancy defects and functionalization by heteroatom [16] and metal doping [17]. In particular, heteroatom doping was effective on the increase of active sites and regulation of electron distribution [18,19,20,21,22]. It has been demonstrated that co-doping multi elements with different electronegativity can remarkably improve the performance of graphene due to the positive synergistic effect of heteroatoms [23,24,25,26,27,28,29,30,31,32,33,34]. Ci et al. [35] simultaneously doped graphene with nitrogen and loaded cobalt to obtain Co/NG catalyst by heat treatment. On the one hand, Co/NG has a relatively large active surface area and high conductivity, which can accelerate the electron transfer rate; on the other hand, catalytic sites such as doped nitrogen and cobalt nanoparticles cooperate to catalyze glucose oxidation and oxygen reduction reaction.

The purpose of this research was to develop a low-cost and high-performance DGAFC anode catalyst. In this study, an N, S and transition metal M (Ni, Co, Fe) co-doped rGO (rGO-NS-M) was synthesized via a simple in-situ hydrothermal synthesis approach. The morphology, chemical composition and defect degree were characterized by SEM, XPS and Raman. Electrochemical performance and fuel cell tests were carried out in a direct glucose alkaline fuel cell (DGAFC) under ambient conditions.

## 2. Experimental

### 2.1. Materials

Graphite powder (Purity 99.95%; 325 mesh Qingdao jinrilai graphite Co., Ltd., Qingdao, China), H_2_SO_4_, HCl, H_2_O_2_ (30%), 60 wt % PTFE solution, activated carbon (AC YEC-8A Fuzhou Yihuan Carbon Co., Ltd., Fuzhou, China), Ni (NO_3_)_2_·6H_2_O, Co (NO_3_)_2_·6H_2_O, Fe (NO_3_)_3_, glucose and KOH were all analytical grades, purchased from the chemical platform of Tianjin University (Tianjin Yuanli Chemical Co., Ltd., Tianjin, China). Nickel foam (average pore diameter of 590 μm, thickness of about 1.7 mm, purity of 99.9%, porosity of 110) was purchased from Changsha Liyuan New Materials Co., Ltd., Changsha, China. Deionized water was used for all the experimental solutions.

### 2.2. Synthesis of rGO-NS

First, the modified Hummer method [36] was used to synthesize the graphite oxide (GO). It was stored under refrigeration and sonicated for another half an hour before use. The rGO-NS was prepared by a simple one-step in-situ hydrothermal approach using GO and thiourea as the precursors. Typically, 1.2 g, 3.6 g, 12 g, 18 g and 24 g of thiourea were dissolved in 60 mL of 2 mg/mL GO solution suspension respectively in order to produce rGO doped with different amounts of N and S. With the stirring, the solutions gradually changed from clear brown-yellow liquids to viscous liquids. Then they were put into a 100 mL Teflon-lined autoclave at 180 °C for 24 h. Finally, the rGO-NS suspension was centrifuged and placed in a −80 °C refrigerator for 12 h, and then placed in a vacuum freeze dryer for 24 h to obtain the rGO-NS powders (rGO-10NS, rGO-30NS, rGO-100NS, rGO-150NS and rGO-200NS).

### 2.3. Preparation rGO-NS-M Catalyst

**Figure 1:**
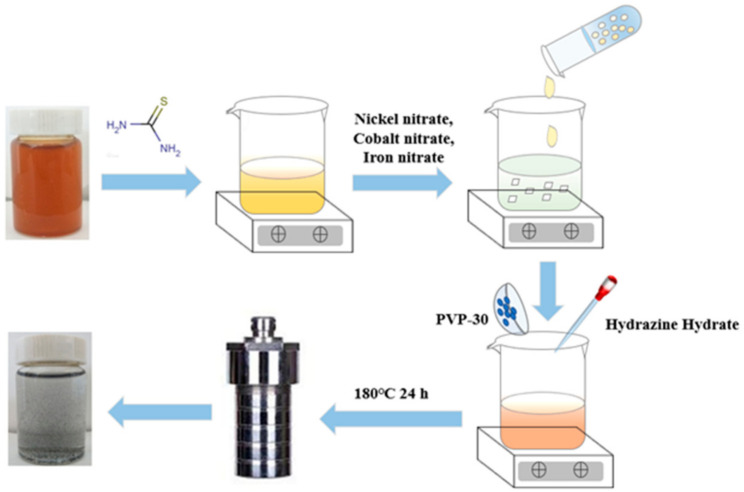
Schematic illustration of the formation process of rGO-NS-M catalyst.

Figure 1 shows the schematic illustration of the preparation process of rGO-NS-M catalyst. The dispersed 6 mg/mL graphite oxide was mixed with 12 g thiourea. The clear brown-yellow graphite oxide solution gradually became viscous. 20 mL of 2 mM nickel nitrate, cobalt nitrate, and iron nitrate were added to the thiourea-graphite oxide solution, respectively. Then 0.5 g of PVP-30 and 2 mL of hydrazine hydrate were added and stirred for one hour. The turbid brown–yellow liquid gradually become a black body suspension during the stirring process. The mixed solution was transferred to an autoclave and heated in an oven for 24 h to prepare rGO-NS-M. The catalyst was washed with distilled water and centrifuged 3–5 times to remove unreacted thiourea, and then was freeze-dried.

### 2.4. Electrodes Preparation and DGAFC Assembly

The rGO-NS-M modified AC anode was prepared with 50 mg rGO-NS-M and 1 g AC. The air cathode was prepared according to the roll pressing method in our previous report [37]. The half-cell performance was tested in a mixed solution 14 mL of 3 M glucose and 1 M KOH.

### 2.5. Materials Characterization and Electrochemical Analysis

The scanning electron microscope (SEM, Japanese-emission scanning electron microscope SU8200), X-ray photoelectron spectroscopy (XPS, the Escalab 250Xi produced by Thermo Fisher Scientific in the United States); and Raman spectrometer (the RM2000 from Renishaw in the United Kingdom) were used for the characterization of nanocomposites. For Raman characterization, a 532 nm laser was used for excitation, and the spectral range was 200 cm^−1^~2250 cm^−1^.

Electrochemical characterizations were carried out by using a rotating disk electrode (RDE) made of glass carbon (GCE, RDE-3A, 3 mm diameter) connected to an electrochemical workstation (CHI 660E, CHI Instrument Co., Ltd., Shanghai, China) and a RRDE-3A (ALS, Tokyo, Japan). Conventional three electrodes system was employed. For cyclic voltammetry (CV) analysis, catalyst ink was prepared and coated onto the GCE as previously described [38]. The catalyst ink was prepared by mixing 5 mg of the catalyst in 8 mL of 1:1 (v/v) deionized water/ethanol along with 50 μL of nafion solution (5 wt %), and well dispersed by 30 min ultrasonic processing. The CV test was conducted under the conditions of 0.6 M KOH and 0.2 M glucose solution, with a potential range of −0.7 V~0.5 V (vs Hg/HgO), and scan rate of 50 mV/s. Linear sweep voltammetry (LSV) with different disk speeds (200 rpm~3000 rpm) were scanned from −0.6 to 0.05 V (vs. Hg/HgO) at a scan rate of 50 mV/s. The catalyst-coated GCE, the graphite rod and Hg/HgO were used as working electrode, counter electrode and the reference electrode, respectively. For EIS, half-cells were assembled. Electrochemical impedance spectroscopy (EIS) measurements were conducted over a frequency range of 100 kHz to 100 mHz. The amplitude of the disturbance current was 10 mV. Zsimpwin software was used to fit EIS data. The electrochemically active surface area (ECSA) of the catalyst is proportional to the electrochemical double-layer capacitance (C_dl_). For C_dl_, CV curves were measured at scan rates of 20, 40, 60, 80, 100 and 120 mV/s in the non-Faradic region, and C_dl_ (mA/cm^2^) was obtained by plotting the current difference (Δj) relative to the scan rate. The ECSA (normalized by mass) was estimated by the following equation [39].

C_dl_ = d(Δj)/2dV_b_ (1)

ECSA = C_dl_/Cs (2)

where Cs is the specific capacitance of the electrode surface. Generally, C_S_ is in the range of 20–60 μF/cm^2^. In this paper, for the catalyst in 0.6 M KOH, the average C_S_ value used according to the literature is 40 μF/cm^2^ [40]. V_b_ is the scan rate. The power density and polarization curve of fuel cell were measured by external resistance method [41].

## 3. Results and Discussion

### 3.1. Electrochemical Performance of Different Anodes

#### 3.1.1. Catalytic Performance of rGO-NS

**Figure 2:**
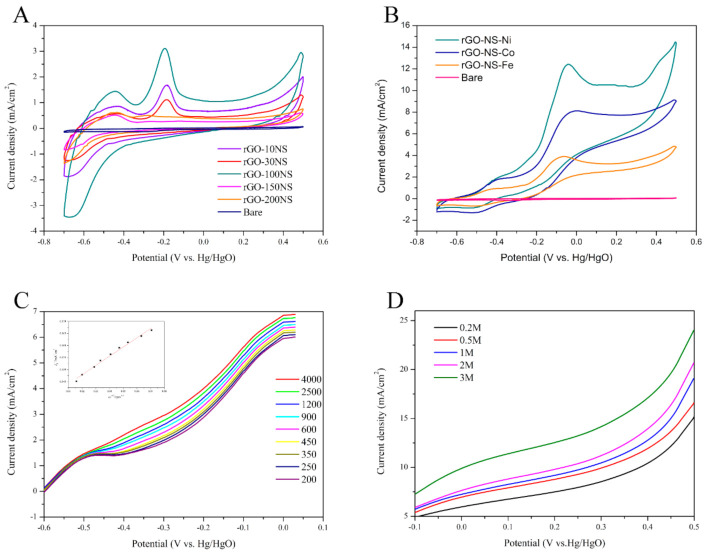
(**A**) Cyclic voltammetry (CV) curves of rGO-10NS, rGO-30NS, rGO-100NS, rGO-150NS, and rGO-200NS in 0.6 M KOH and 0.2 M glucose solution (50 mV/s scan rate, 25 °C). (**B**) CV curve of rGO-NS-M in 0.6 M KOH and 0.2 M glucose solution (**C**) Rotating disk electrode (RDE) curves for glucose solution at 0.2 M on rGO-NS-Ni in 0.6 mol/L KOH solution, at 25 °C. Scan rate: 50 mV/s. (**D**) Linear sweep voltammetry (LSV)curve of rGO-NS-Ni in different concentrations of glucose solution (50 mV/s scan rate, 25 °C).

Thiourea was used as a nitrogen (N) and sulphur (S) doping agent to prepare rGO-NS. It can be speculated that the ratio of thiourea to GO can have impact on the catalyst performance, thus the ratio of thiourea to GO was optimized at first. Figure 2A shows the CV curves of rGO-NS with different doping ratios. There were two main oxidation peaks in the CV curve, appeared around −0.45 V and −0.18 V (vs. Hg/HgO), respectively. These peaks might be due to the removal of hydrogen from hemiacetal hydroxyl group of glucose and the chemical adsorption of hemiacetal (C1) carbon on the catalyst surface. For rGO-10NS, rGO-30NS and rGO-100NS, the oxidation peaks were located at −0.45 V and −0.18 V (vs. Hg/HgO). The peak height gradually increased with the increase of doping ratio of N and S. However, with the continuous increase of doping of N and S (rGO-150NS and rGO-200NS), the height of the peak located at −0.45 V (vs. Hg/HgO) decreased, and the peak position originally at −0.18 V (vs. Hg/HgO) was negatively shifted. The oxidation peaks at −0.18 V (vs. Hg/HgO) were almost disappeared indicating that excessive N and S doping may block or destroy the active sites of the catalyst. As a whole, rGO-100NS possessed the best catalytic performance.

#### 3.1.2. Catalytic Performance of rGO-NS-M

Figure 2B shows there were two oxidation peaks in the CV curves of rGO-NS-M (Ni, Co, Fe). For rGO-NS-Ni and rGO-NS-Co, the first oxidation peak of were located at −0.41 V (vs. Hg/HgO), and the second oxidation peak appeared at −0.044 V (vs. Hg/HgO). For rGO-NS-Fe, the first oxidation peak was negatively shifted to −0.44 V (vs. Hg/HgO), and the second oxidation peak was left shifted to −0.073 V (vs. Hg/HgO) compared with those of rGO-NS-Ni and rGO-NS-Co. These results indicated that the catalytic ability of rGO-NS-Ni was the best, followed by rGO-NS-Co and rGO-NS-Fe.

In order to further study the kinetic process of catalytic glucose oxidation, we measured the LSV curve (scanning speed was 50 mV/s) using RDE and constructed the corresponding Koutecky−Levich (K−L) fitting curve of the catalyst at different speeds (200–3000 rpm) in 0.6 M KOH solution. The electrooxidation of glucose on rGO-NS-Ni produces a similar profile, which persists in all speed ranges. The limiting current obtained on the rGO-NS-Ni electrode increases with the rotation speed of the electrode. The higher the speed, the shorter the diffusion distance, indicating that the process is a diffusion control process. It can be seen from the Figure 2C that the K-L line of the rGO-NS-Ni catalyst shows a good linear relationship below 1000 rpm and the fitted line shows good parallelism, indicating that it was fitted with the first-order reaction kinetics. From the Levich plots, it is found that the linearity at lower speed was due to the limiting current controlled by mass transfer. It deviates from linearity at higher speed, indicating that there was a dynamic limit.

LSV curves of rGO-NS-Ni in different concentrations of glucose varying between 0.2–3 M in 0.6 M KOH were measured under room temperature at the scanning speed of 50 mV/s. Figure 2D shows the effect of glucose concentration on current density. It can be seen from LSV that the current density increases to a considerable extent with the increase of glucose concentration. When the electrode potential was 0.5 V (vs. Hg/HgO), the current density of rGO-NS-Ni anode in 3 M glucose reached 23.99 mA/cm^2^, which is 1.58 times of the current density of 0.2 M (15.12 mA/cm^2^).

**Figure 3:**
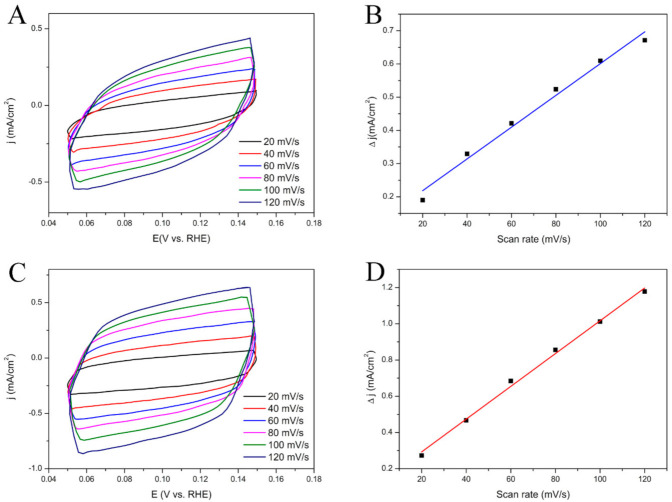
(**A**) CV curves of (**A**) rGO-100NS and (**C**) rGO-NS-Ni with different scan rates from 20 to 120 mV/s in the range of 0.05–0.15 V in 0.6 M KOH. Scan rate dependence of the current densities of (**B**) rGO-100NS and (**D**) rGO-NS-Ni.

In order to understand the enhanced glucose oxidation reaction performance of the rGO-NS-Ni in comparison to that of rGO-NS, their electrochemically active surface areas (ECSAs) were estimated by collecting CV curves (Figure 3). The calculated value of ECSA for the rGO-NS-Ni was ~60.05 m^2^/g, significantly higher than that of rGO-100NS (~31.71 m^2^/g), indicating that Ni doped rGO-100NS (rGO-NS-Ni) has more active reaction sites.

#### 3.1.3. The Performance of rGO-NS-M Anode

**Figure 4:**
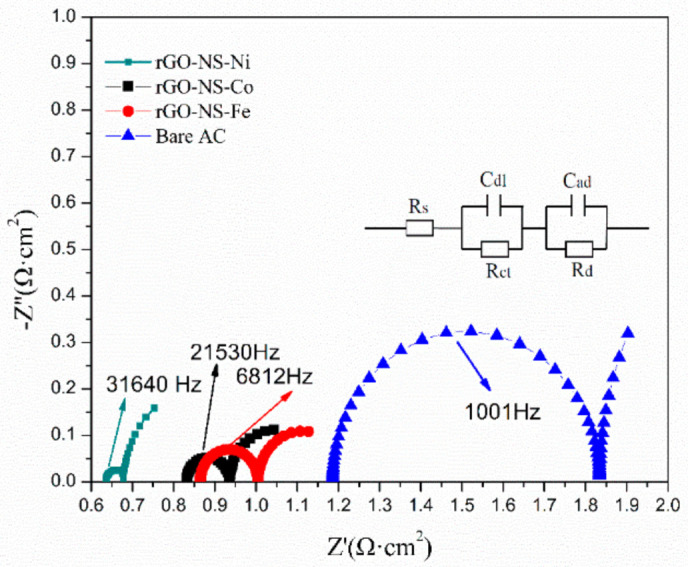
Electrochemical impedance spectroscopy (EIS) curve of rGO-NS-M anode.

By measuring impedance changes in a wide frequency range, EIS can get important information on electrode reaction kinetics and electrode interface structure. Figure 4 shows the Nyquist curves of all electrodes modelled by the equivalent circuit (R (CR) (CR)). The equivalent circuit diagram (the embedded diagram of Figure 4) was plotted to fit the EIS. In this equivalent circuit, the total resistance (Rt) was composed of five parts: diffusion resistance (Rd), pore adsorption capacitance (Cad), charge transfer resistance (Rct), layer capacitance (Cdl) and ohmic resistance (Rs). The changes in resistance of each anode were reflected in Table 1 and Figure 4. It can be seen from Table 1 that the addition of the rGO-NS-M had remarkably decreased the resistance of the electrodes. Rt of each anode changed in an order of: Bare AC (3.3814 Ω) > rGO-NS-Fe (1.4519 Ω) > rGO-NS-Co (1.3867 Ω) > rGO-NS-Ni (1.1584 Ω). rGO-NS-Ni anode showed the lowest resistance among the four anodes, which was 2.92 times lower than that of Bare AC. The resistance data of the ohmic resistance (Rs) was observed. Since each test used an electrolyte with the same composition under the same conditions, the ohmic resistance values corresponding to different anodes have no obvious changes. The total resistance was a combination of Rs, Rct and Rd. The change in total resistance can be attributed to the changes in these three types of resistance. The laws of Rs, Rct, and Rd were consistent with Rt. Table 1 also indicated that both Rct and Rd contributed to the reduction of Rt. The Rd of rGO-NS-Ni was nearly 3.71 times lower than that of Bare AC. This value means that the addition of rGO-NS-Ni makes the anode diffusion resistance (Rd) greatly reduced compared to rGO-NS-Co and rGO-NS-Fe. The addition of nickel greatly contributes to the diffusion of the matrix.

### 3.2. DGAFC Performance

**Figure 5:**
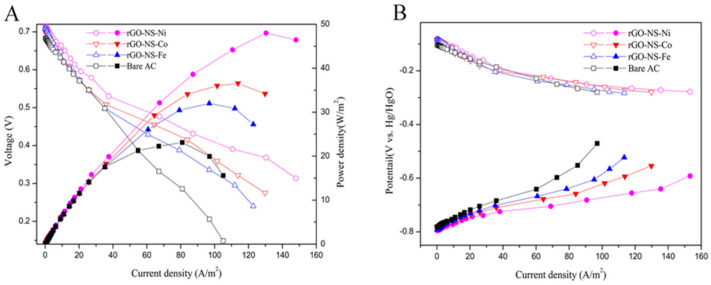
(**A**) Power density curves of the direct glucose alkaline fuel cell (DGAFC) equipped with different rGO-NS-M anodes. (**B**) Polarization curves of cathode and different anodes.

Figure 5A showed that the power density curves of DGAFC equipped with different graphene-NS-M anodes. The maximum power density of each experimental group in descending order was rGO-NS-Ni (48.0 W/m^2^) > rGO-NS-Co (36.56 W/m^2^) > rGO-NS-Fe (31.99 W/m^2^) > Bare group (Bare AC) (23.12 W/m^2^). The maximum power density of rGO-NS-Ni was nearly 2.08 times that of the blank group. The order for the maximum current density was rGO-NS-Ni (148.0 A/m^2^) > rGO-NS-Co (129.74 A/m^2^) > rGO-NS-Fe (122.83 A/m^2^) > Blank group (Bare AC) (105.10 A/m^2^). All these results clearly demonstrated that rGO-NS-Ni has boosted the fuel cell performance.

In order to further explore the reasons for the increase in power density and current density, the cathode and anode polarization curves of DGAFC equipped with different rGO-NS-M anodes were measured (Figure 5B). The trends of the cathode polarization curves of fuel cells were consistent confirmed the difference on fuel cell performance came from anode. For the anode polarization curve, the RGO-NS-M with the largest power density and current density had the smallest anode polarization. The degree of anode polarization was in the order of blank group (Bare AC) > rGO-NS-Fe > rGO-NS-Co > rGO-NS-Ni. This was consistent with the results of power density and current density tests. It confirmed that the boosted performance of power density and current density was due to the low polarization of the anode. Table 2 shows the performance of the recently published literature. Compared with those noble metal electrochemical catalysts, rGO-NS-M showed excellent performance.

### 3.3. Characterization of rGO-NS-Ni Nanocomposites

**Figure 6:**
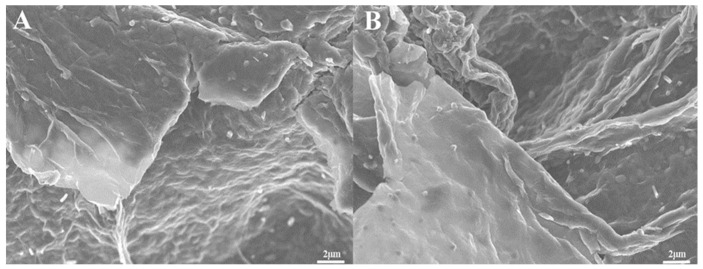
SEM image (**A**,**B**) of rGO-NS-Ni composite with the magnifications were 5 k.

In Figure 6A,B, it can be seen that some small particles were uniformly distributed on the surface of graphene. These particles should be Ni particles in the rGO-NS nanocomposite. rGO-NS may have played an auxiliary role in the uniform distribution of nickel in the synthesis of rGO-NS-Ni. It displayed a soft and smooth surface and complex wrinkles. The high surface area of rGO-NS-Ni was beneficial to the catalytic process in the fuel cell. During the electrocatalytic process, increased surface area results in faster chemical reactions due to the increase of exposure of active sites to the substrates. At the same time, this multi-corrugated surface was conducive to the storage of electrons, thereby increasing the overall capacitance. In a word, the surface morphology of rGO-NS-Ni was considered responsible for the performance improvement. It was believed that this surface morphology can increase the exposed active sites, prevent agglomeration of Ni nanoparticles, and boost the transfer of electrons.

**Figure 7:**
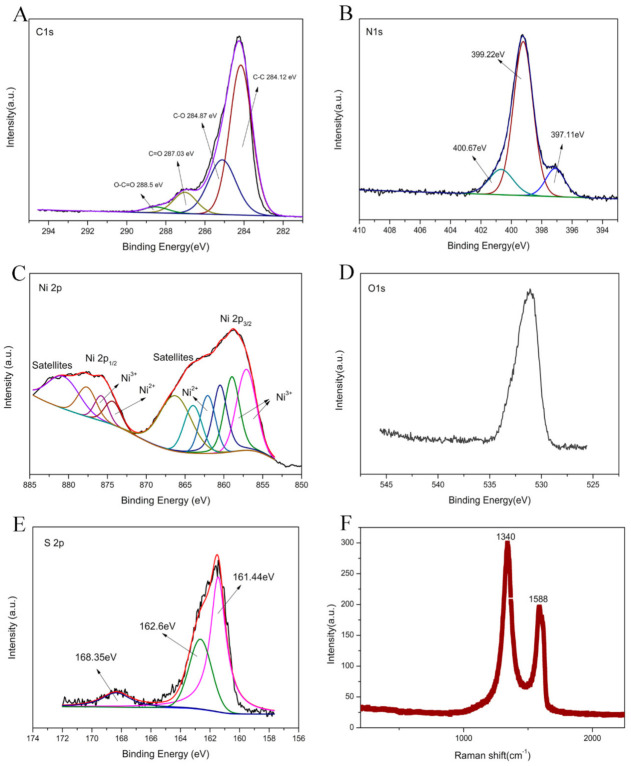
XPS spectrum of rGO-NS-Ni electrode: (**A**) C 1 s spectrum; (**B**) N 1 s spectrum; (**C**) rGO-NS-Ni 2 p spectrum; (**D**) O 1 s spectrum; (**E**) S 2 p spectrum; (**F**) Raman spectrum of rGO-NS-Ni.

In order to investigate the existence state of each element in rGO-NS-Ni, and probe the catalytic mechanism in DGAFC, XPS spectra were measured (Figure 7A–E). In C1s spectrum, there were four peaks, which correspond to non-oxidized carbon C-C (284.12 eV), carbon atom in C-O group (284.87 eV, epoxy or hydroxyl), carbonyl carbon C=O (287.03 eV), and carboxyl carbon O-C=O (288.5 eV), respectively. The non-oxidized carbon C-C accounted for the highest proportion obviously. In addition to non-oxidized carbon, there were carbonyl carbons, carboxyl carbons, and other oxidized carbons, indicating that pristine GO existed in rGO-NS-Ni. The existence of a small amount of GO in rGO was reasonable due to the uncompleted reduction. In the N 1S spectrum of the XPS (Figure 7B), rGO-NS-Ni had three peaks, corresponding to pyridine nitrogen (397.11 eV), pyrrole nitrogen (399.22 eV) and graphite nitrogen (400.67 eV), respectively. The structure of these nitrogen elements in rGO-NS-Ni was that the pyridine nitrogen combines with two carbon atoms, and one of the p electrons was located in the p conjugated system so that the pyrrole nitrogen is accompanied by a pair of p electrons [47]. Graphite nitrogen represents nitrogen atoms that replace carbon atoms in the hexagonal ring in graphene [48]. Figure 7C shows the details of high-resolution spectrum of Ni 2p, which could be fitted into two states peaks. For Ni2p_3/2_ and Ni2p_1/2_, binding energies were placed at 858.73 and 857.87 eV ± 0.2 eV, respectively. The pair of binding energies existed at 857.11, 858.97 and 857.78 eV were attributed to Ni^3+^, and the other pair at the energies of 874.31 and 862.09 eV was corresponding to Ni^2+^. They represent Ni^3+^ in NiOOH and Ni^2+^ in Ni-O and Ni (OH)_2_, respectively. In addition to these five peaks, the remaining five peaks were all satellite peaks, and their corresponding binding energies were 860.5, 880.67, 866.24, 863.97 and 877.64 eV, respectively. In the S2p spectrum (Figure 7E), there were two types of S species located at 162.6 eV and 161.44 eV, with a splitting energy of 1.16 eV, corresponding to C-S-C and -C=S-, respectively. The strong peak near 168.35 eV can also be attributed to some oxidized sulfur, which usually forms at the edge of graphene. Due to the spin-orbit coupling in the sulfur atom, the first two peaks were related to the 2p_3/2_ (161.44 eV) and 2p_1/2_ (162.6 eV) of thiophene S [42]. The remaining peak C-SOx-C was caused by the sulfur element in sulfur oxide.

Raman spectra illustrated the defective degree of as-prepared rGO-NS-Ni (Figure 7F). The absorption peaks of rGO-NS-Ni mainly appear at 1340 cm^−1^ and 1588 cm^−1^. These two peaks are mainly related to graphene. The content of Ni element in the rGO-NS-Ni catalyst relative to graphene was less than four thousandth of graphene and the content of nitrogen and sulfur doped was very small. Therefore, it was not detected due to the detection limits of Raman. The G band, which is the peak at 1590 cm^−1^, was ascribed to the carbon sp^2^ vibration of the in-plane domain in the material. While the D-band, which is the peak at 1340 cm^−1^, came from the out-of-plane vibration mode, indicating the presence of sp^3^ carbon in the carbon material. The ratio of the intensity of the D band to the G band (ID/IG) was the key to distinguish graphene and graphite oxide. By reducing the oxygen-containing functional groups in GO, a large number of small-sized sp^2^ conjugated domains were formed, resulting in a reduction in the average size of the planar sp^2^ domains. In general, when ID/IG < 1, the main content of the material was GO. When ID/IG > 1 in the Raman spectrum, rGO mainly existed in the material. The ID/IG ratio of rGO-NS-Ni was close to 2 indicated that GO had been reduced to rGO, and there were many edges and defects presented in rGO-NS-Ni. The increased edges and defects should be due to the chemical modification of N and S, which interrupt the uniform π system of graphene and introducing more point defects [49].

### 3.4. Feasible Mechanism

**Figure 8:**
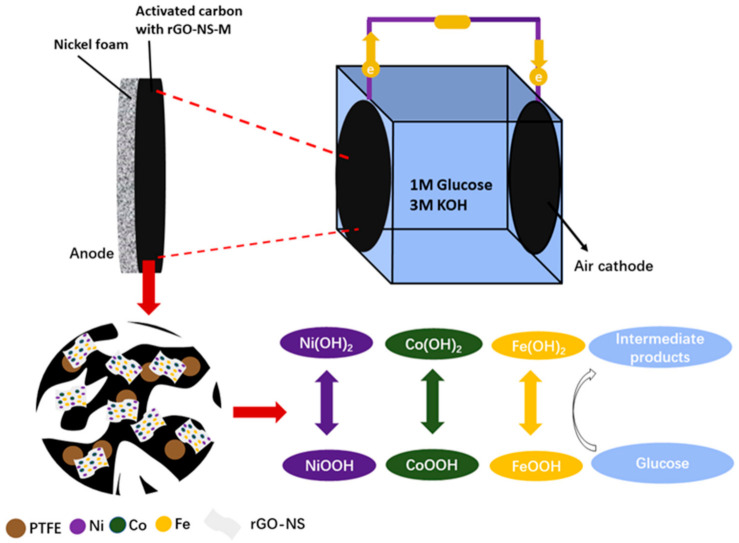
Schematic diagram of the catalytic mechanism for rGO-NS-M involved in glucose oxidation and power generation.

For DGAFC, the first anodic reaction was thought to be the oxidation of glucose by hydroxide (OH^−^) to produce glucolactone and release electrons. The anode collected the electrons and output them to the external circuit. On the cathode, oxygen captured these electrons and reacted with water to regenerate OH^−^. For rGO-NS-M, both transitional metals and N, S-doped rGO may attend the anodic oxidation processes. According to the XPS results, NiOOH was deduced to be the intermediate products in the catalytic oxidation process. NiOOH can capture the electrons from glucose and were converted into Ni (OH)_2_. NiOOH can be regenerated from Ni (OH)_2_, after their reaction with OH^−^, which continue to join the catalytic oxidation process so that the cycle can continue (Equations (3) and (4)). We speculate that Co and Fe follow the same reaction rule as Ni (Equations (5)–(8)). Therefore, the catalytic effect of metal elements can be attributed to the redox couples of Ni^2+^/Ni^3+^, Co^2+^/Co^3+^ and Fe^2+^/Fe^3+^. During their redox cycle, oxidation of glucose was enhanced and electricity generation was boosted (Figure 8).

(3) $${\mathrm{Ni}(\mathrm{OH})}_{2}{+\mathrm{OH}}^{-}{-e}^{-}\to {\mathrm{NiOOH}+H}_{2}O$$

(4) $$\mathrm{NiOOH}+\mathrm{glucose}\to \mathrm{Ni}{\left(\mathrm{OH}\right)}_{2}\;+\mathrm{glucolactone}$$

(5) $${\mathrm{Co}(\mathrm{OH})}_{2}{+\mathrm{OH}}^{-}{-e}^{-}\to {\mathrm{CoOOH}+H}_{2}O$$

(6) $$\mathrm{CoOOH}+\mathrm{glucose}\to \mathrm{Co}{\left(\mathrm{OH}\right)}_{2}\;+\mathrm{glucolactone}$$

(7) $${\mathrm{Fe}(\mathrm{OH})}_{2}{+\mathrm{OH}}^{-}{-e}^{-}\to {\mathrm{FeOOH}+H}_{2}O$$

(8) $$\mathrm{FeOOH}+\mathrm{glucose}\to \mathrm{Fe}{\left(\mathrm{OH}\right)}_{2}\;+\mathrm{glucolactone}$$

In addition to the redox couple of the metal elements, the doped nitrogen and sulfur heteroatoms in rGO-NS-M should also played an important role. Studies have shown that inorganic heteroatoms such as nitrogen and sulfur can change the electronic properties and chemical reactivity of graphene [50,51,52,53]. XPS detected the presence of the chemical bond C=C-N (graphite nitrogen) in rGO-NS-Ni, which can create more edges and defects on rGO. Nitrogen and sulfur can covalently bonded to rGO with pyrrole nitrogen, pyridine nitrogen and thiophene sulfur, or coexist with rGO in a non-covalent bond. These structures can provide a larger electrolyte/electrode interface, thus resulting in a smaller interface resistance. The key problem in the glucose oxidation process at the DGAFC anode was the suppressed electron transfer process. The doping of N, S and transitional metals in rGO accelerated the electron transfer and created more active sites for electrochemical catalysis, so that rGO-NS-M has a better catalytic ability.

## 4. Conclusions

N, S and transition-metal co-doped rGO nanocomposites were successfully synthesized by a simple one-step hydrothermal approach. The catalytic ability of the nanocomposites on glucose oxidation was evaluated in a DGAFC. rGO-NS-Ni had the best electrochemical performance with the maximum power density of 48.0 W/m^2^, which was 2.08 times higher than that of the Bare AC (23.12 W/m^2^). The maximum current density was 148.0 A/m^2^, which was increased by 40.82% compared to the Bare AC (105.10 A/m^2^). It is speculated that the high performance of rGO-NS-M was due to the increased conductivity and active sites on rGO by doping with N, S, and transitional metals. Furthermore, the redox couples of Ni^2+^/Ni^3+^, Co^2+^/Co^3+^ and Fe^2+^/Fe^3+^ significantly promoted the catalytic oxidation of glucose. The low-cost and high-performance rGO-NS-M nanocomposites have great potential for large-scale applications, which can promote the application of DGAFC.

## Figures and Tables

**Table 1:** Fitted resistance values of different rGO-NS-M anodes.

Composites	Rs (Ω)	Cad (F)	Rct (Ω)	Cdl (F)	Rd (Ω)	Rt (Ω)
Bare AC	1.1860	0.0003	0.6484	3.2390	1.5470	3.3814
rGO-NS-Fe	0.8645	64.2800	0.2287	0.0020	0.1400	1.4519
rGO-NS-Co	0.8318	19.3900	0.2254	0.0298	0.1041	1.3867
rGO-NS-Ni	0.6833	67.4400	0.2153	0.5486	0.0445	1.1584

**Table 2:** Comparison of power generation performance of DGAFCs equipped with different anodes.

Anode Catalyst	Maximum Power Density (W/m^2^)	Glucose (M)	T (°C)	Noble Metal	Compart-Ments	Catalyst Loading	Peak Current Density (mA/cm^2^)	Reference
AuPt/rGO	11.860	0.05	-	Yes	Two	0.19 mg/cm^2^	-	[42]
Ni-Co-rGO	28.807	1	25	No	One	0.15 mg/cm^2^	-	[43]
Ni_3_N-Co_3_N	30.89	1	25	No	One	0.15 mg/cm^2^	-	[44]
Pd/N-3D mesoporous carbon-	-	0.01	36.6	Yes	-	0.36 mg/cm^2^	2.5	[45]
Pd-Ce_2_O_3_/ITO	-	0.5	25	Yes	-	-	8.0	[46]
rGO-NS-Ni	48.00	3	25	No	One	0.15 mg/cm^2^	-	This work
rGO-NS-Co	36.56	3	25	No	One	0.15 mg/cm^2^	-	This work
rGO-NS-Fe	31.99	3	25	No	One	0.15 mg/cm^2^	-	This work
